# Recurrent Syncope as Initial Symptom in Apical Intrathoracic Tumor

**DOI:** 10.4021/jocmr765w

**Published:** 2012-01-17

**Authors:** Hung Yi Chen

**Affiliations:** a Department of Cardiology, Taipei City Hospital- Heping Branch, Taiwan

## Abstract

Syncope is a common complaint in clinical medical care. The etiology and mechanism can be complex. We report two cases experienced recurrent syncope to our emergency room without concomitant symptom. The image studies of both cases revealed apical neoplasms compress left common carotid artery with mediastinal lymphadenopathy and without cardiac/main artery trunk involved. After brain lesion, cardiac arrhythmia, and orthostatic hypotension were excluded, syncope related to vagus nerve involved was suspected.

**Keywords:**

Recurrent syncope; Intrathoracic tumor; Neurocardiogenic

## Introduction

Syncope is a common complaint in clinical medical care. Except neurological disorder, most of the patients are a brief benign clinical course with spontaneously recovery. The etiology may be range from benign disorders to severe life-threatening disease. In spite of extensive evaluation, to identify the underlying etiology remains difficult in some cases. Intrathoracic neoplasm manifesting as recurrent syncope is rare and the majority of such cases are mediastinum related with cardiac or pulmonary artery involved. We report two cases with intrathoracic neoplasm who present with cardiac but without respiratory symptoms. Being an uncommon manifestation, we described the two cases and discussed the possible mechanism.

## Case Report

### Case 1

**Figure 1:**
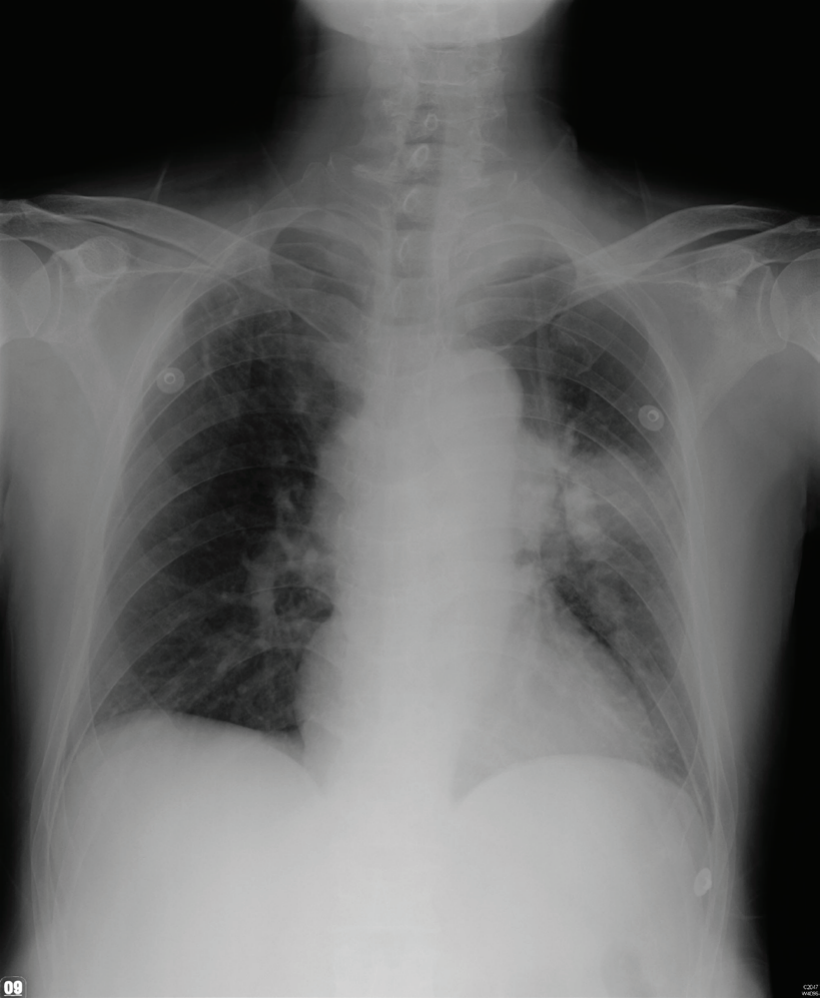
Chest radiography on emergency room shows a soft tissue density mass at left upper lobe with prominent soft tissue density at left apex and left lower neck.

**Figure 2:**
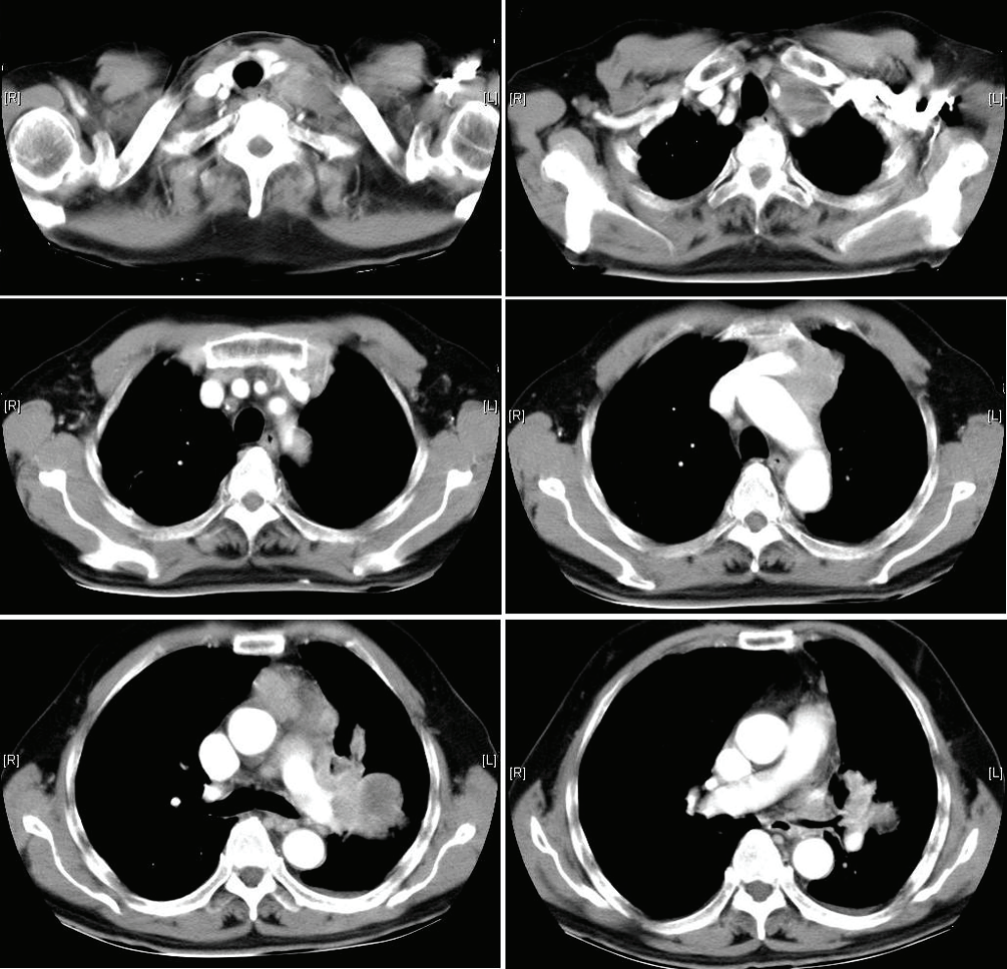
Chest CT (with contrast) demonstrates a 4.5 cm soft tissue mass with heterogenous enhance at left lower lobe with lymphadenopthy at left hilum, left paraaortic, anterior mediastinum and left upper paratracheal site (lower and middle panels). The lymphadenopathy extends to left lower neck and causes obstruction of left upper lobe bronchus, right side displacement of trachea, thyroid gland. Left carotid artery and internal jugular vein are compressed and the latter is not opacified by contrast medium.

A 73-year-old man was admitted to our emergency department with complaining of repeated syncope in two days. Prodromal symptoms of light headache and diaphoresis prior to the episodes were told. He regained consciousness spontaneously followed by brief conscious loss without mental impairment. The events occurred on his toilet in the fist episode. The second event developed on the following day when he was sitting and watching television. With similar warming symptoms as light headache and diaphoresis, the brief loss of consciousness was followed by full recovery. He was otherwise well, and had no chest pain, shortness of breath, or palpitation before the episodes. He also experienced a recent unproductive cough, but not concomitant with syncope. He had neither systemic disease as hypertension nor medical history before and without medication used. He had a history of smoking for decades and quit for many years without drinking. His blood pressure (BP) was 118/72 mmHg and pulse rate was 64 beats/minute on arrival. No significant abnormalities were discovered at the time of physical examination of heart and lung. There was no abnormal finding in neurologic examination. Brain computed tomography (CT) was checked and the results showed normal study without vascular atherosclerosis change. His electrocardiogram (ECG) revealed sinus rhythm and unremarkable. Biochemical analysis demonstrated normal cardiac enzyme (troponin I, total creatine kinase and CK-myocardial isoenzyme) level. Chest radiography was checked and showed a soft tissue density mass at left upper lobe. There was prominent soft tissue density at left apex and left lower neck (Fig. 1). He was advised to be admitted to our hospital under the suspicion of pulmonary tumor. On the following day after admission, chest CT was arranged and performed. The results showed a 4.5 cm soft tissue density mass with heterogenous enhancement at left lower lobe near hilum with extensive lymphadenopathy at left hilum, left paraaortic, anterior mediastinum with left paratracheal extending to left lower neck. The lymph nodes caused obstruction of left upper lobe bronchus with right side displacement of trachea, thyroid gland, and left internal jugular vein was compressed (Fig. 2). Pulmonary neoplasm with lymphadenopathy was suspected and the patient was discharged for the secondary opinion after we explained the probability with his family.

### Case 2

**Figure 3:**
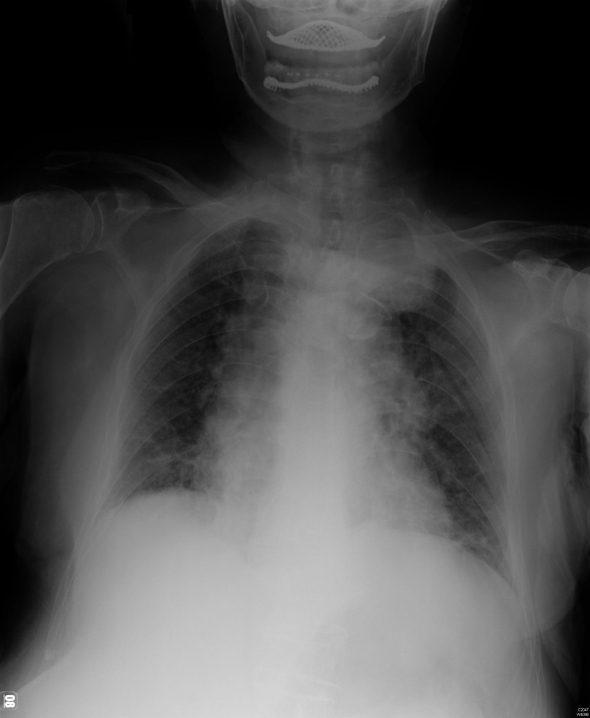
Chest radiography shows a soft tissue lesion at left apex.

**Figure 4:**
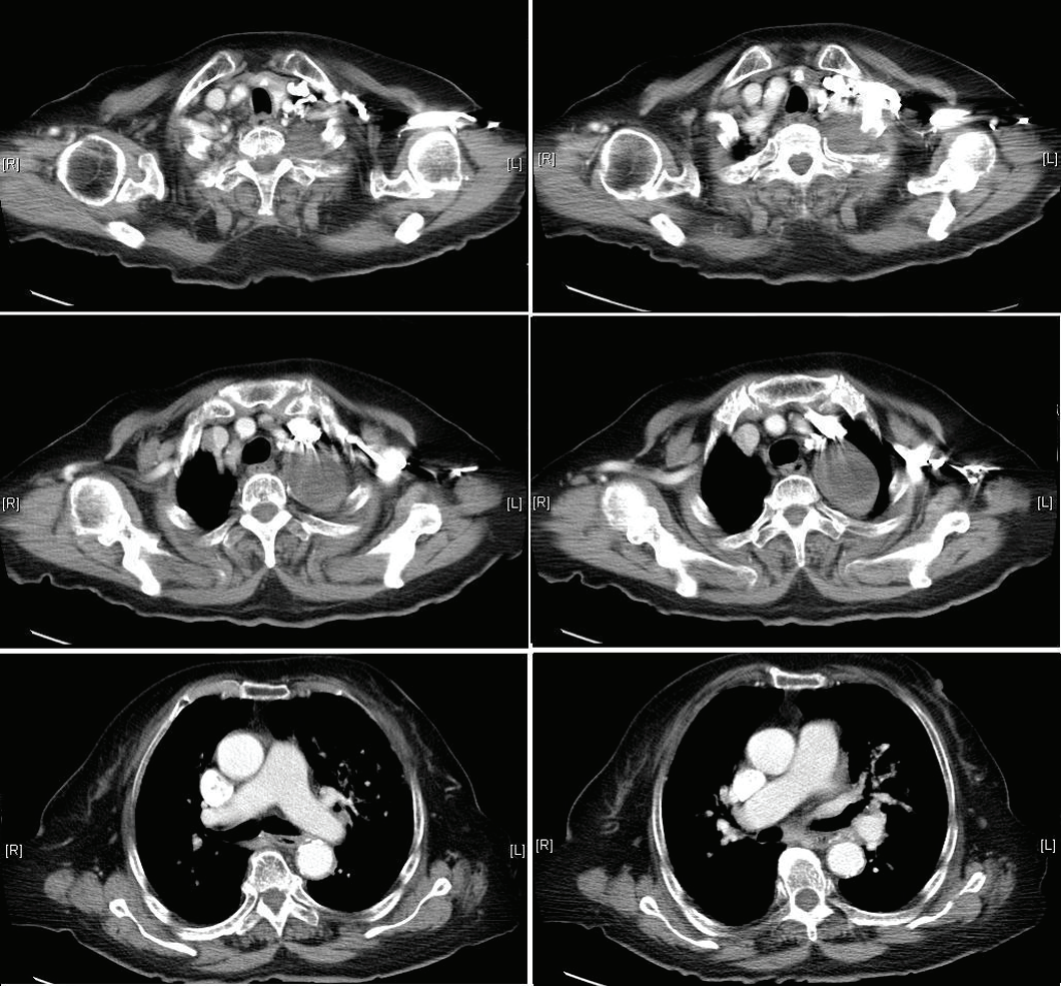
Chest CT (with contrast) demonstrates a 4.0 cm soft tissue mass at left lung apex compressing the left common carotid artery without compromise of blood flow. There are several oval lymph nodes at mediastinum.

A 82-year-old female was admitted to our emergency department with complaining of sudden conscious loss for minutes with spontaneous recovery for two episodes in a morning. All the incidences had occurred when she was on ordinary activity. In each occasions, she experienced visual field darkness initially, then with cold sweating and vertigo and finally syncope. There was no chest pain, shortness of breath, or palpitation before the episodes. She had a medical history of diabetes without medication. She had no history of smoking and alcoholic consumption. On physical examination, her BP was 124/61 mmHg and pulse rate was 87 beats/minute. Chest radiography disclosed an enlarged cardiac silhouette and air-space consolidation in at both lung fields and a 5 x 3 cm soft tissue lesion at left apex (Fig. 3). A 12-lead ECG demonstrated sinus rhythm without ST-segment and T-wave abnormalities. Laboratory investigation revealed mild-degree anemia and high sugar level (hemoglobin 12.2 g/dL, blood-sugar 196 mg/dL) and normal cardiac enzyme levels. All remaining laboratory examinations were normal. After admission, brain CT was arranged and it showed neither abnormal intracranial hemorrhage nor density change except brain atrophy. Twenty-four-hour Holter ECG was checked for arrhythmia related syncope and the results showed sinus rhythm with scattered atrial/ventricular premature beats. And chest CT demonstrated a 4.0 x 5.5 x 5.5 cm soft tissue tumor at left lung apex with several lymph nodes at mediastinum (Fig. 4). After explained and discussed the patient’s condition with her family, they decided to discharge without further evaluation due to her old age.

## Discussion

Syncope is a common complaint in clinical medical care. Excluding neurological disorder, the majority of patients with syncope are related to neurocardiogenic. It is defined as the transient loss of blood flow to the brain, secondary to hypotension after excessive vasopressor reflex. Drop in blood pressure caused by bradycardia (or asystole) results in transient conscious loss. Most of the conscious loss last for seconds to minutes with spontaneously recover and present as a benign clinical course. Episodes of such syncope mostly occur in the standing or sitting positing position and are associated with nausea, dizziness, or diaphoresis. Although the abnormal reflex responsible for neurcardiogenic syncope is believed, the possible underlying pathophysiologic mechanism remains complicated and poor understood. There are several theories suggested to explain the pathophysiology of neurocardiogenic syncope [1]. Because various mechanisms may contribute to the cause in different patients or even simultaneously in one patient, the cause of neurocardiogenic syncope is occasionally difficult to determine. Even extensive examination and evaluation in patient with syncope is undertaken, to identify the underlying etiology sometimes remains a challenge. This leads to a diagnostic dilemma for which a wide range of possibilities needs to be considered.

Syncope as a symptom in patients with intrathoracic neoplasm is uncommon. The majority of the cases present with mediastinal tumor and the cause of syncope is related to pulmonary arteries or cardiac involvement. Pulmonary embolism, cardiac arrhythmia, or pulmonary artery hypertension can be the possible mechanisms [2]. A reduction of cardiac output by decreased venous return could also induce syncope [3]. Although with large occupied lesion at left hilum in case 1, this is no evidence in our patients with cardiac or pulmonary trunk invasive by image data. In addition to cardiac involved, syncope as a result of malignancy had been documented in head and neck cancer. Glossopharyngeal syncope associated with tumor of the oropharynx or base of the skull had been demonstrated [4]. Similar vasovagal effect in intrathoracic neoplasm had been reported in a case of B-cell lymphoma caused a significant compromise of the right internal jugular vein and encasing the right internal carotid artery, narrowed its caliber and induced recurrent syncope [5]. Stimulation to the hypopharyngeal nerve by tumor mass results in activation of the vagus nerve and induces transient cardioinhibition and vasodepression. Neurally mediated syncope can be directly induced by an intrathoracic neoplasm stimulating the vagus nerve [6]. The stimulation leading to syncope by malignancy can be either mechanical or chemical stimulation [7-9]. In our individuals, there were no definite mechanisms proven to cause syncope. However, lacking of the probable etiologies as organic brain lesion, epilepsy, cardiovascular disease, cardiac arrhythmia, orthostatic hypotension, and metabolic encephalopathy, the potential mechanism of recurrent and episodic neural mediated syncope could be caused by apical intrathoracic tumor involving upper rootlets of the left vagus nerve [10,11].

Our cases suggest recurrent syncope could be the first symptom of an intrathoracic neoplasm. Although we can not obtain definite diagnosis by pathological study and lack of their outcome follow-up because both of them refused further evaluation and management. In view of this experience, we suggest the newly developed neurocardiogenic syncope should alert the possibility of malignancy when history taking and clinical examination indicated heart cause were unlikely, and the mechanism did not favor emotional stress triggered.
